# Effect of herbal extracts and supplement mixture on alcohol metabolism in Sprague Dawley-rats

**DOI:** 10.1007/s13197-022-05580-4

**Published:** 2022-09-14

**Authors:** Hyeonjeong Choe, Injue Yun, Yunyoung Kim, Ji-Heon Lee, Hyun-A. Shin, Yong-Kyu Lee, Mi-Yeon Kim

**Affiliations:** 1 Natural FNP Co., Ltd., Yongin, 16827 Korea; 2 Korea National University of Transportation, Chungju, 27469 Korea

**Keywords:** Herbal extract, Hangover, Alcohol dehydrogenase, Aldehyde dehydrogenase, Detoxification

## Abstract

This study aimed to investigate the effect of mixture of herbal extracts and supplementary formula (FNP-C) on hangovers and antioxidant enzymes in alcohol-induced liver damage in rats. HepG2 cells were used as the experimental cells and divided into five groups: non-treated control (normal), alcohol-induced control (control), mixture of herbal extracts (FNP-B), FNP-C, and a commercial treatment of liver diseases (Livers^®^); inhibition of detoxification and alcohol-induced damage was confirmed in vivo. Blood alcohol and acetaldehyde concentration after alcohol consumption were measured in a timely manner; alcohol dehydrogenase (ADH), aldehyde dehydrogenase (ALDH), superoxide dismutase (SOD), glutathione (GSH), glutathione transferase (GST), and lactate dehydrogenase (LDH) levels were measured in the liver. FNP-C exhibited the highest effect. When FNP-C was administered to alcohol-induced animals, blood alcohol and acetaldehyde concentration decreased compared to FNP-B and Livers^®^. FNP-C reduced ADH levels and improved LDH, GSH, GST, and SOD levels. The FNP-C group was effective in preventing alcohol-induced hangovers and liver damage. Thus, FNP-C improves hangovers and increases antioxidant activity in an alcohol-induced model. Adding amino acids and vitamins to natural ingredients can potentially enhance the effect of improving hangovers.

## Introduction

Hangovers are characterized by headache, tremulousness, nausea, diarrhea, and fatigue, along with decreased occupational, cognitive, or visual–spatial skill performance. In the United States, related absenteeism and poor job performance cost $148 billion annually. Hangovers are associated with alcoholism, but most of its costs occur in light-to-moderate drinkers (Wiese et al. 2000).

Alcohol is rapidly metabolized to acetaldehyde by alcohol dehydrogenase (ADH), which is then metabolized to acetic acid by aldehyde dehydrogenase (ALDH) in the liver (Lieber 2004). Consuming large amounts of alcohol produces excess acetaldehyde by ADH. Acetaldehyde induces damages onto hepatocytes and neurocyte. Furthermore, it stimulates sympathetic nerve and vagus nerve fibers to induce the core symptom of hangover. In addition, reactive oxygen species (ROS) is generated by the microsomal ethanol oxidizing system and catalase in the process of alcohol metabolism and also is one of the main causes of aging and disease (Lieber 1999).

To counter these destructive processes, organisms have developed extensive enzymatic and non-enzymatic systems to prevent and recover from ROS-mediated damage. The major antioxidant systems are superoxide dismutase (SOD), glutathione S-transferase (GST), glutathione peroxidase (GPx), glutathione reductase (GR), and catalase (CAT) (Matés et al. 1999). There is a balance between ROS and antioxidants, which is essential for the health of an organism (Zakhari 2006).

Plants, fruits, and vegetables are abundant in antioxidants, such as polyphenolic components, isoflavonoids, and vitamins, which can scavenge free radicals. Among them, *Pinus densiflora* cv., *Alnus japonica* cv., *Eleutherococcus senticosus* cv. (Choi et al. 2006), *Panax ginseng* cv*.* Berry, *Opuntia humifusa* cv., *Hovenia dulcis* cv., *Pueraria lobata* cv., and *Camellia sinensis* cv. can suppress the adverse effects of alcohol exposure (Na et al. 2018; Kim et al. 2004; Wiese et al. 2004). In addition, vitamins and amino acids such as zinc, nicotinic acid, vitamin B6, taurine, tryptophan, and betaine have been reported to be essential for alcohol metabolism (Dougherty et al. 2007; Jung et al. 2013; Kägi and Vallee 1960; Verster et al. 2019). They have also been reported to alleviate alcohol-induced oxidative stress by stimulating the activity of antioxidant enzymes (Jung et al. 2013).

In a previous study on *a pilot case* in human, FNP-B of 2.7 g helped the decrease in the breath alcohol concentration (BAC) after alcohol intake (Hwang and Kim 2020). It is needed to determine the effect on the rate of alcohol and acetaldehyde reduction and the difference whether the supplements like zinc, nicotinic acid, etc. are added or not in vivo model.

Therefore, eight herbal extracts were prepared and this formulation was used to measure the effect of alcohol and acetaldehyde-oxidizing pathways on changes in blood alcohol and alcohol metabolism enzyme levels in alcohol-treated animals. Additionally, the effect was measured by the addition of nutrients for hangover-relieving effects in an acute alcohol-administered rat model.

## Materials and methods

### Materials

FNP-B was composed with 8 kinds of herbal extracts reported to the synergistic effects when blended in ratio (Hwang and Kim 2020), which are shown in are listed in Table 1. NFP-ADH-03 composed with *Alnus japonica*, *Pinus densiflora* and *Eleutherococcus senticosus* was extracted with water at 100 °C and then evaporated to dryness under reduced pressure. Herb extracts of *Camellia sinensis*, *Pueraria lobate*, *Panax ginseng* Berry, *Hovenia dulcis* Fruit, and *Opuntia humifusa* were purchased separately (Table 1).

**Table 1:** Composition of herbal extracts and supplement formula

Section	Material name	Formulation ratio (%)	Supplier
Herbal extracts	NFP-ADH-03	57.91	Haram Co., LTD, Korea
	*Alnus japonica*	36.84	
	*Pinus densiflora*	36.84	
	*Eleutherococcus senticosus*	26.32	
	*Camellia sinensis*	14.55	Roasting house Co., LTD., Korea
	*Pueraria lobata*	14.55	Haram Co., LTD, Korea
	*Panax ginseng.* Berry	7.72	Saerom epeuaenbi Co., LTD, Korea
	*Hovenia dulcis.* Fruit	4.83	ShinWoo Co., LTD, Korea
	*Opuntia humifusa*	0.44	Prndle Co.,LTD, Korea
	Total	100.00	
Supplement formula	Betaine	71.60	Shinwoo CO., LTD, Korea
	Vitamin C	10.25	Mireu International CO.,LTD., Korea
	Taurine	10.25	Mireu International CO.,LTD., Korea
	Nicotinic acid amide	5.11	Seongu BIO Pam Co.,Ltd, Korea
	Vitamin B6	1.53	Mireu International CO.,LTD., Korea
	Zinc Oxide	0.76	Sewon Coporation, Korea
	l-tryptophan	0.50	Unigen Inc, Korea
	Total	100.00	

FNP-C was formulated with FNP-B and 7 non-herbal supplements known to ameliorate alcohol induced oxidative stress, which are listed in Table 2. Seven supplements are zinc oxide, nicotinic acid amide, vitamin B6, vitamin C, taurine, betaine, and l-tryptophan, which were purchased.

**Table 2:** Experimental design

Groups	Ingredients and dose
FNP-B	Herbal extract 150 mg/kg: NFP-ADH-03, *Camellia sinensis*, *Pueraria lobata*, *Panax ginseng.* Berry, *Hovenia dulcis.* Fruit, *Opuntia humifusa*
FNP-C	Herbal extract 150 mg/kg: NFP-ADH-03, *Camellia sinensis*, *Pueraria lobata*, *Panax ginseng*. Berry, *Hovenia dulcis*. Fruit, *Opuntia humifusa* Supplement formular 80 mg/kg: Betaine, Vitamin C, Taurine, Nicontinic acid amide, Vitamin B6, Zinc oxide, L-tryptophan
Livers^®^	405 mg/kg l-Arginine, Betaine, Betaine hydrochloride, Citric acid hydrate

The Liver’s Sol^®^ (Livers^®^), a commercial treatment of liver diseases, was used as a positive control which was composed of L-arginine (36.10%), betaine (24.69%), betaine hydrochloride (24.69%), and citric acid hydrate (14.52%).

### Cell culture and treatment

Human hepatoma (HepG2) cell line was purchased from the American type culture collection (ATCC 8065, ATCC, USA). HepG2 cells were cultured in Dulbecco’s modified Eagle’s medium (DMEM, Hyclone, USA) supplemented with 10% fetal bovine serum (FBS, Gibco, USA) and 1% penicillin/streptomycin (P/S, Gibco, USA) in a humidified atmosphere containing 5% CO_2_ in air at 37 °C. HepG2 cells were incubated in the presence or absence of different concentrations of FNP-B, FNP-C (100–800 ug/ml) or Livers^®^ (0.05–0.4%) and then the hepatotoxicity was stimulated by the addition of ethanol (4.5%) for 24 h.

### Cell viability assay

Cell viability was determined by MTT(3-[4,5-dimethyl-thiazol-2-yl]-2,5-diphenyl tetrazolium bromide) assay as described previously (Ciapetti et al. 1993). In brief, HepG2 cells were seeded in 96 well plate at a density of 3 × 10^4^ cells/well. Cells were pre-treated with FNP-B, FNP-C and livers for 2 h and then stimulated with ethanol at concentration 4.5% for 24 h. Subsequently, 20 uL of MTT solution (1.67 mg/ml) was added to each well and incubated at 37 °C for 2 h. After 2 h incubation, medium was removed and dissolved with 200 uL of dimethyl sulfoxide (DMSO). Absorbance was measured at 550 nm using the SpectraMax^®^ M3 Multi-Mode Microplate reader (Molecular Devices, USA) and the percentage of cell viability was calculated.

### Animals

Sprague Dawley rats (male, 6-week-old and 200–220 g body weight) were purchased from DBL (Eumsung, Korea). Animals were housed under 12 h light/dark cycles and at 22 ± 2 °C and were acclimatized for 7 days. They are feed with standard diet and water ad libitum. The rats were randomly divided into five groups of seven rats in each group. FNP-B group was administered 150 mg/kg of FNP-B. FNP-C group was administered 230 mg/kg of FNP-C, which is a mixture of 150 mg of FNP-B and 80 mg of 7 supplements (Table 2). Livers^®^ group was administered 405 mg/kg, with the human equivalent dose (HED) using the body surface area. In a previous study, 2.7 g of complex extracts of FNP-B was administered and its hangover relieving effect was verified in human (Hwang and Kim 2020). Normal group and control group were orally given saline. After 30 min, ethanol was administered 2.4 g/kg by i.p. to all groups except non-alcohol group. This study was approved (KNUT IACUC 2021-1) by Institutional Animal Care and Use Committee (IACUC) of Korea national university of Transportation.

### Measurement of alcohol and acetaldehyde level in blood

In order to measure the alcohol and acetaldehyde level in blood by time, blood was collected through caudal vein for 0.25, 0.5, 1, 3, and 7 h after alcohol administration. The collected blood was centrifuged at 3000×*g* for 15 min to separate the serum. Separated serum was used for measuring the contents by Ethanol assay kit (K-ETOH, Megazyme, USA) and Acetaldehyde assay kit (K-ACHYD, Megazyme, USA). Change of the absorbance at 340 nm for 3 min was calculated as the amount of NADH.

### Determination of liver damage by different assay system

Rats were sacrificed at 7 h from alcohol administration and the liver was dissected out. The isolated liver was rapidly frozen in liquid nitrogen, stored in a − 70 °C until analysis, and washed with phosphate-buffered saline (PBS) before analysis. Liver homogenate was mixed with each assay buffer of ADH activity kit (K787-100, Biovision, USA), ALDH activity kit (K731-100, Biovision, USA), lactate dehydrogenase (LDH) activity kit (K726-500, Biovision, USA), and GST activity kit (K263-100, Biovision, USA). After mixing, the supernatant was used for measuring the absorbance by a SpectraMax^®^ M3 Multi-Mode Microplate Reader (Molecular Devices, USA).

### Measurement of GSH content

In order to evaluate the GSH contents of liver in rats, the dissected liver at 7 h after alcohol administration was homogenized with 5% of sulfosalicylic acid solution and obtained a supernatant from which the nucleus and unground parts were removed. The GSH content was measured by using a GSH assay kit (K464-100), and the absorbance was measured at 450 nm using a SpectraMax^®^ M3 Multi-Mode Microplate Reader (Molecular Devices, USA) and expressed in mmol/mg.

### Statistics

The results were expressed in mean ± standard deviation (SD). Statistical analysis was performed with Student’s *t* test. The significance was determined at *p* < 0.05.

## Results

### Cell viability of HepG2 cells

**Fig. 1:**
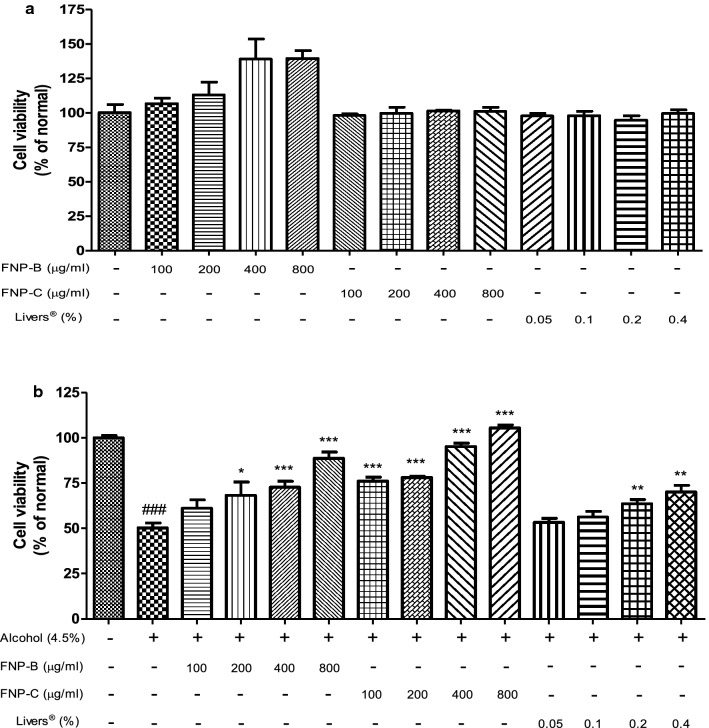
Protective effect of FNP-B and FNP-C on hepatoma HepG2 cells treated with alcohol. Data are expressed means ± standard deviations (n = 5 times). Percentage of viable cells was calculated with normal cell. ^###^*p* < 0.001 vs Normal cell. *p < 0.05; **p < 0.01; ***p < 0.001 vs Alcohol treated cell

Prior to the hepatoprotective studies, the cytotoxic effects of FNP-B and FNP-C were examined in human hepatoma HepG2 cells. Cells were incubated with various concentrations of FNP-B, FNP-C (100, 200, 400, and 800 µg/mL), and Livers^®^ (0.05, 0.1, 0.2, and 0.4%) for 24 h. MTT analysis showed no significant difference in cell viability (Fig. 1a), indicating no cytotoxicity in HepG2 cells.

After treatment with 4.5% ethanol, relative cell viability was reduced significantly by approximately 50% (Fig. 1b). However, the relative viability of HepG2 cells treated with FNP-B, FNP-C, and Livers^®^ was higher than that of the normal cells. The relative cell viability of the treated group increased in a dose-dependent manner compared to that of the normal group. The median effective concentration (EC_50_) was derived for each group; the EC_50_ of FNP-B, FNP-C, and Livers^®^ were 475, 65, and 937 µg/mL. FNP-C had the lowest EC_50_, which was shown to be an effective formulation for cell viability, and a synergistic protective effect against ethanol-induced stress was observed in the HepG2 cells.

### Concentration of alcohol and acetaldehyde in blood

**Fig. 2:**
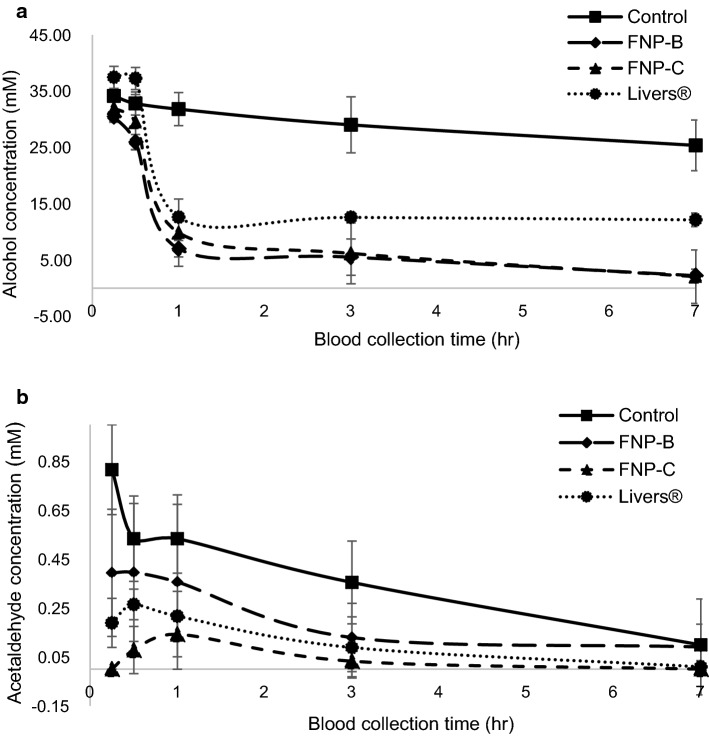
Change of alcohol and acetaldehyde concentration in blood for 7 h in SD rat administrated with FNP-B and FNP-C. Data are expressed as mean ± SD

FNP-B. FNP-C, Livers^®^, and saline were orally administered 30 min before alcohol administration. Alcohol and acetaldehyde levels in the blood were determined at 0.25, 0.5, 1, 3, and 7 h after alcohol administration. Alcohol concentration in control group peaked 0.25 h (34.12 ± 3.59 mM) and gradually decreased thereafter. The alcohol concentration in the three treated groups exhibited a significant decrease compared to the control groups at 0.5 h after alcohol administration (Fig. 2a and Table 3). The blood alcohol concentration was found to be 12.62 ± 0.83 mM (Livers^®^), 7.01 ± 5.98 mM (FNP-B), and 9.87 ± 1.46 mM (FNP-C) at 1 h. When the alcohol reduction rate was compared with the initial dose, the reduction percentages at 1 h were 66% (Livers^®^), 69.2% (FNP-C), and 76.7% (FNP-B). In the Livers^®^ group, the alcohol reduction rate did not increase even at 3 and 7 h, but in FNP-B and FNP-C, the alcohol reduction percentage increased to 92–93% over time. FNP extracts composed of eight herbs tended to continue to decompose alcohol over time compared to Livers^®^, which is composed of betaine, L-arginine, and citric acid. This result is consistent with that for alcohol degradation, which was not better promoted in FNP-C than in FNP-B. These supplements had no significant effect on alcohol degradation.

**Table 3:** Concentration of alcohol in blood at time

Section	Time	Control	FNP-B	FNP-C	Livers®
Alcohol concentration (mM)	0.25	34.12 ± 3.59	30.46 ± 2.33*	31.97 ± 0.99	37.45 ± 1.98
	0.5	32.79 ± 2.06	25.95 ± 4.91**	29.47 ± 1.33**	37.24 ± 1.98*
	1	31.79 ± 2.94	7.01 ± 5.98***	9.87 ± 1.46***	12.62 ± 0.83***
	3	29.01 ± 4.98	5.52 ± 5.41***	6.19 ± 3.22***	12.58 ± 0.87***
	7	25.36 ± 4.49	2.22 ± 4.74***	2.05 ± 1.15***	12.14 ± 1.20***
Acetaldehyde concentration (mM)	0.25	0.82 ± 0.18	0.39 ± 0.26*	0.00 ± 0.00***	0.19 ± 0.10***
	0.5	0.53 ± 0.17	0.40 ± 0.28	0.08 ± 0.10***	0.26 ± 0.06**
	1	0.53 ± 0.14	0.36 ± 0.36	0.14 ± 0.09***	0.22 ± 0.10**
	3	0.35 ± 0.17	0.13 ± 0.14*	0.03 ± 0.06**	0.09 ± 0.12*
	7	0.10 ± 0.08	0.09 ± 0.20	0.00 ± 0.00*	0.01 ± 0.03*

Data are expressed as mean ± SD (n = 7 rats). *p < 0.05; **p < 0.01; ***p < 0.001 vs Control group. Statistical significance was tested with the *t* test

The blood acetaldehyde concentration also peaked at 0.25 h after alcohol administration (0.82 ± 0.18 mM) in the normal group (Fig. 2b and Table 3). The blood acetaldehyde concentration was maintained at 0.53 mM between 0.5 and 1 h after alcohol administration. In the other groups, blood acetaldehyde was detected 0.25 h after alcohol intake, but its levels were significantly lower than those in the control group. All groups exhibited higher acetaldehyde concentrations at 0.5 and 1 h than acetaldehyde concentration at 0.25 h. The time period in which the alcohol concentration decreased the most was between 0.5 and 1 h, and because of this, the concentration of acetaldehyde was maintained at 0.5 to 1 h or showed a tendency to increase. Blood acetaldehyde concentrations were significantly lower than those in the control group in the order of FNP-B > Livers^®^ > FNP-C at every collection time (Table 3). FNP-C exhibited a significantly low acetaldehyde concentration.

### Effect of treatment on ADH and ALDH activities

**Fig. 3:**
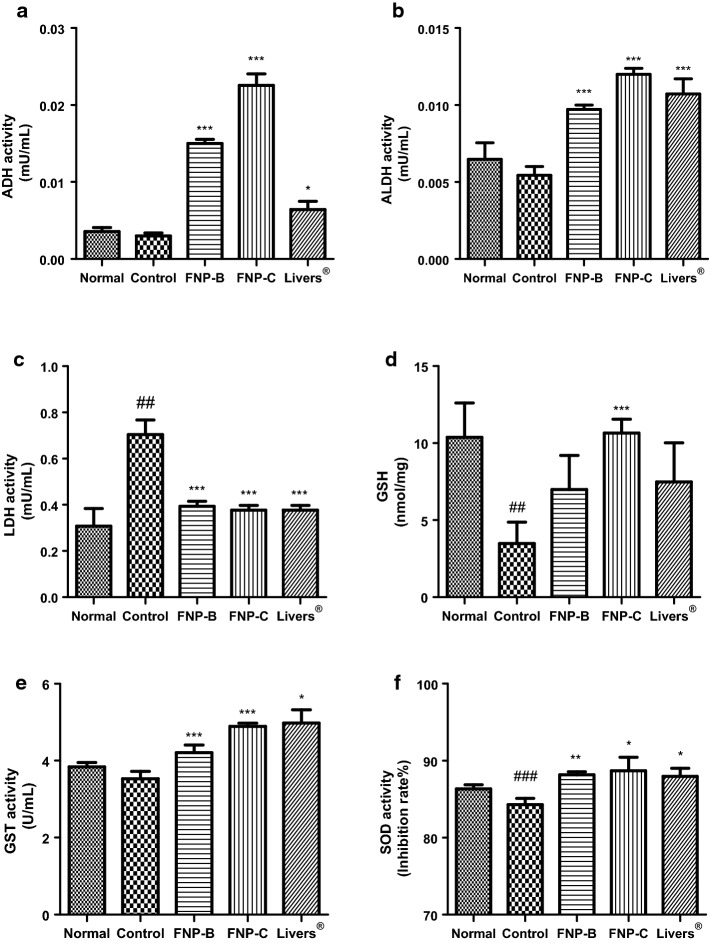
Effect of FNP-B and FNP-C on Hangover related enzyme activity in liver. ADH activity (**a**), ALDH activity (**b**), LDH activity (**c**), GSH content (**d**), GST activity (**e**) and SOD activity (**f**) liver at 7 h after alcohol administration in SD rat. Data are expressed as mean ± SD of 7 rats. ^#^*p* < 0.05 vs normal group. *p < 0.05, **p < 0.01 and ***p < 0.001 vs Control group. Statistical significance was tested with the *t* test

The activities of ADH and ALDH were investigated to examine the liver activity after 7 h of alcohol treatment. ADH and ALDH activities were lowest in the control group. However, an increase in ADH level was observed in all treatment groups, and the efficacy was highest in the order of FNP-C (0.023 ± 0.004 mU/mL), FNP-B (0.015 ± 0.001 mU/mL), and Livers^®^ (0.007 ± 0.003 mU/mL; Fig. 3a). Similarly, the activity of ALDH was significantly increased in all treatment group. It was highest in the order of FNP-C (0.012 ± 0.001 mU/mL), Livers^®^ (0.011 ± 0.003 mU/mL), and FNP-B (0.010 ± 0.001 mU/mL) compared with control group (0.006 ± 0.002 mU/mL; Fig. 3b).

### Effect of treatment on LDH activity

The LDH activity in the liver of SD rats 7 h after alcohol administration is shown in Fig. 3c. The LDH activity of the control group (0.70 ± 0.17 mU/mL) was significantly increased compared to the normal group (0.31 ± 0.20 mU/mL). The activity of LDH was significantly decreased in FNP-B (0.39 ± 0.06 mU/mL), FNP-C (0.38 ± 0.05 mU/mL), and Livers^®^ (0.27 ± 0.17 mU/mL). No increase in LDH levels in the liver due to alcohol intake was observed in any of the treatment groups.

### Effect of treatment on GSH content and SOD and GST activities

The GSH content and activities of the antioxidant enzymes GST and SOD in the liver tissues were measured (Fig. 3d–f). The hepatic antioxidant enzyme-related activities of GST, GSH, and SOD decreased after alcohol administration.

The contents of GSH was 10.66 ± 2.38 nmol/mg (FNP-C), 7.00 ± 5.85 nmol/mg (FNP-B), and 7.48 ± 6.72 nmol/mg (Livers^®^; Fig. 3d). Activity of GST was found to be 4.89 ± 0.22 U/mL (FNP-C), 4.21 ± 0.54 U/mL (FNP-B), and 4.98 ± 0.91 (Livers^®^; Fig. 3e). The SOD activity of the FNP-B, FNP-C, and Livers^®^ groups was significantly increased with values of 88.17 ± 1.03%, 88.68 ± 4.68%, and 87.95 ± 2.79%, respectively, compared to the control group (Fig. 3f).

Pretreatment with FNP-B, FNP-C, and Livers^®^ significantly prevented the depletion of antioxidant enzymes from alcohol intake.

## Discussion

In all the groups, alcohol concentration similarly decreased by 0.2–4.5 mM during the initial 30 min, but the alcohol level of the treated groups decreased significantly compared to the control between 0.5 and 1 h. The alcohol reduction rate was compared with the initial dose, and the reduction percentages at 1 h were 6.9% (Control), 66.4% (Livers^®^), 69.2% (FNP-C), and 76.7% (FNP-B). FNP-B and FNP-C showed similar activity. In the Livers^®^, which is composed of betaine, L-arginine, and citric acid, the alcohol concentration did not decrease even after 1 h, which is the same tendency as that of the control. The alcohol reduction percentage increased to 92–93% as time passed in FNP-B and FNP-C. FNP-B extracts composed of eight herbs tend to continue decomposing alcohol over time. Non-herbal supplements had a significant effect on alcohol degradation after 3, 7 h of alcohol ingestion.

In this experiment, the peak time of blood acetaldehyde after alcohol intake was between 0.5 and 1 h, which is consistent with the report of Iess et al. (Lee et al. 2012). In the control group, the alcohol content decreased from 34.12 to 32.79 mM and acetaldehyde content decreased from 0.82 to 0.53 mM within 0.5 h of alcohol intake. At 0.25 h, 0.82 mM of acetaldehyde was produced, and an additional 0.25 h later, the concentration of alcohol decreased by 2 mM and that of acetaldehyde decreased to 0.53 mM in blood. The alcohol level was further decreased by 2 mM between 0.5 and 1 h, but acetaldehyde levels did not increase, showing a tendency to be maintained. These results showed that the herbal extracts effectively reduced acetaldehyde in the blood. In addition, supplements show to help effectively the decomposition of alcohol and acetaldehyde on the decomposition of alcohol and acetaldehyde when it mixed with herbal extract.

The hepatic activities of ADH and ALDH are affected by alcohol administration and increased by plant extracts (Lee et al. 2020). Our results demonstrated that hepatic ADH activity after 7 h of alcohol intake was decreased in the control group but increased fivefold in the FNP-B group, sevenfold in the FNP-C group, and twofold in the Livers^®^ group. ADH activity was the lowest in the Livers^®^ group, which tended to coincide with the highest blood alcohol concentration of Livers^®^ at 7 h. Hepatic ALDH activity was also decreased by alcohol administration but increased twofold in the FNP-C and Livers^®^ groups and 1.4-fold in the FNP-B group. ALDH activity was relatively lower in the FNP-B group and these results were consistent with those of studies that showed that the blood acetaldehyde level was relatively high in the FNP-B group. Based on these results, FNP-C which was formulated with herbal extracts and supplement formula had an excellent effect on the decomposition of alcohol and acetaldehyde by increasing ADH and ALDH enzyme activity. Various elimination pathways for alcohols and acetaldehydes may be helpful for alcohol hangovers. Polyphenolic compounds are reported to decrease the levels of alcohol and acetaldehyde in the blood via ADH and ALDH (Deng et al. 2013; Fu et al. 2011; Lee et al. 2012). In the liver, the activation of SOD, GST, and GSH plays an important role in prevent oxidative damage caused by alcohol radicals.

The increased LDH activity in alcohol-treated rats may indicate increased permeability, damage, and necrosis of hepatocytes (Liu et al. 2010). The control group showed a significant increase in LDH activity compared to the normal group, but LDH activities in the FNP-B and FNP-C groups were similar to those of the normal group. Non-herbal supplements of taurine (Wu et al. 2009), zinc (Caglar et al. 2012), and vitamins (Sönmez et al. 2012) was reported to be effective in decreasing LDH and FNP-C also contains these components.

Twenty percent of alcohol absorbed into the body undergoes absorption and decomposition in the stomach; the rest of the absorption occurs in the small intestine and moves into the blood and finally broken down in the liver (Lee et al. 2016). When a large amount of alcohol is consumed in a short period, excessive amounts of acetaldehyde and NADH are produced by ADH. Alcohol that has not been decomposed in the blood or liver generates oxygen radicals, which combine with glutathione and cysteine to form lipid peroxide in the plasma. It denatures the cell membrane, ultimately leading to liver damage (Lee et al. 2016). Furthermore, long-term ingestion of alcohol not only damages the liver but also causes fatal damage to metabolic processes in the body.

A hangover that appears after excessive drinking is a physical and psychological phenomenon that includes nausea, vomiting, dizziness, and headaches. Rapid removal of alcohol and acetaldehyde from the blood will help to reduce the hangover. Alcohol breakdown in the liver results in the formation of molecules, leading to ROS production in which further metabolism in the cell causes cytotoxicity (Sung et al. 2016; Wu and Cederbaum 2003). The hangover-relieving effect of FNP-B and FNP-C using the optimal mixing ratio demonstrated hepatoprotective effects in HepG2 cells. Also, in previous study, antioxidant effect of mixture of herbal extracts in vitro has been verified (Hwang and Kim 2020). Although it does not reflect uptake metabolism in vitro, hepatoprotective and antioxidant effects were also verified in vivo.

In the in vivo study, blood alcohol and acetaldehyde concentrations were significantly decreased by increasing ADH and ALDH activity in the liver tissue in the FNP-B and FNP-C groups. In addition, it is suggested that FNP-B and FNP-C have hepatoprotective effects by improving the antioxidant defense system, such as GSH, GST, and SOD levels.

Based on these results, FNP-B and FNP-C would be useful functional formulations for hangover care. In particular, the combination of herbal extracts and non-herbal supplements has been shown to be an important factor in enhancing the hangover relieving effect by activating antioxidant enzymes to remove radical, and improving the alcohol metabolism rate by stimulating alcohol metabolizing enzymes.

## Data Availability

Not applicable.
